# High levels of serum superoxide dismutase as a biomarker of intrahepatic cholestasis of pregnancy in patients with viral hepatitis B

**DOI:** 10.1186/s12884-022-04776-y

**Published:** 2022-05-28

**Authors:** Fei Wang, Yajuan He, Naijuan Yao, Litao Ruan, Zhen Tian

**Affiliations:** 1 Department of Ultrasound, The First Affiliated Hospital of Xi’an Jiaotong University, Shaanxi province Xi’an City, China; 2 Department of Infectious Diseases, The First Affiliated Hospital of Xi’an Jiaotong University, Shaanxi province Xi’an City, China

**Keywords:** Intrahepatic cholestasis of pregnancy, Hepatitis B virus, Oxidative stress, Superoxide dismutase

## Abstract

**Background:**

Intrahepatic cholestasis of pregnancy (ICP) is characterized by skin pruritus and impaired liver function. Hepatitis B virus (HBV) infection increases the risk of developing ICP. HBV infection is associated with oxidative stress, which has been proven to participate in the development of ICP. The goal of this study was to explore the relationship among HBV, oxidative stress, and ICP, and investigate whether a biomarker of oxidative stress may predict the diagnosis and severity of ICP.

**Methods:**

We induced a retrospective cohort of 70 ICP patients from January 2019 to December 2020, and compared their data with those from healthy pregnant women (*n* = 70). Serum levels of an oxidative stress marker superoxide dismutase (SOD) were examined using an enzyme-linked immunosorbent assay (ELISA). Diagnostic and prognostic values of serum SOD were analyzed by receiver operating characteristic (ROC) curve.

**Results:**

Pregnant women in the ICP group had significantly higher level of serum SOD (243.24 ± 12.57 U/L vs 98.70 ± 2.95 U/L, *p* < 0.01) and a higher rate of HBV infection (51.53% vs 25.71%, *p* < 0.05) compared with the control group. HBsAg-positive ICP patients had a higher levels of serum SOD (287.24 ± 19.21 U/L vs 196.65 ± 11.75 U/L, *p* < 0.01) compared with HBsAg-negative ICP patients. A serum SOD level > 121.4 U/mL might be used to predict ICP, while a serum SOD level > 274.6 U/mL might predict ICP severity.

**Conclusion:**

HBV infection promotes oxidative stress during the pathogenesis of ICP. Serum levels of SOD could be used to predict ICP diagnosis and severity. Modification of oxidative stress might be a treatment target for ICP.

## Background

Intrahepatic cholestasis of pregnancy (ICP) is a prevalent liver disorder and affects 1%-4% of pregnant women across the world. ICP is a pregnancy-related condition that manifests itself as otherwise unexplained pruritus, abnormal liver function tests, and elevated serum total bile acid (TBA) levels, especially in the third trimester [1]. The etiology of ICP is not fully explained, genetic, endocrine, and environmental factors are considered to have a role in the disease pathogenesis of ICP [2]. It has been reported that approximately 2–4% of ICP pregnancies result in fetal mortality. The level of TBA increases the risk of fetal morbidity and stillbirth. Despite the use of various agents, ursodeoxycholic acid (UDCA) is the most effective agent in the treatment [3].

In developing nations, hepatitis B virus (HBV) infection has become one of the most common public health problems. In China, the prevalence of HBV infection among women of childbearing age ranges from 2 to 8% [4]. HBV infection has been linked to an increased risk of ICP, although the underlying mechanism has not yet been discovered [5, 6]. Clarifying the relationship between HBV infection and ICP development during pregnancy might help researchers to better understand the pathophysiology of ICP and provide new markers for ICP diagnosis and therapy.

Several studies have shown that HBV infection is associated with oxidative stress, and oxidative stress has been demonstrated to have a role in the development of ICP [7, 8]. Superoxide dismutase (SOD) converts harmful superoxide to harmless hydrogen peroxide, thereby limiting the negative consequences of oxidative stress. In a recent investigation, we discovered that blood SOD levels were higher in patients with liver failure, and that serum SOD levels were linked to the disease severity [9, 10]. Here, we evaluated the link between HBV infection, oxidative stress, and ICP, as well as the diagnostic and prognostic values of serum SOD in ICP.

## Material and methods

### Patients

A total of 70 individuals with ICP, including 36 ICP patients with chronic hepatitis B (CHB) infection, were included in the study from January 2019 to December 2020, at the First Affiliated Hospital of Xi’an Jiaotong University, Shaanxi, China. All of the participants provided written informed consent, and the study was approved by the Research Ethics Committee of the First Affiliated Hospital of Xi’an Jiaotong University.

Before collecting blood samples, no ursodeoxycholic acid was given. The diagnostic criteria for ICP and for recruiting and excluding subjects were as follows. ICP was diagnosed in pregnant women presenting with classical pruritus associated with abnormal liver biochemistry indexes, including TBA, alanine aminotransferase (ALT) and aspartate aminotransferase (AST). Among them, the cutoff level of TBA was 10 μmol/L. Individuals with other causes of liver dysfunction, including preeclampsia, HELLP (hemolysis, elevated liver enzymes, and low platelets) syndrome, acute fatty liver of pregnancy, primary biliary cirrhosis, viral hepatitis, and any ultrasound abnormality that may result in biliary obstruction were excluded. Those with multiple pregnancies, chromosomal abnormalities, fetal anomalies, maternal infection, itching with elevated liver enzymes and normal bile acids, and pruritus with skin lesions were also excluded. A total of 70 age-matched healthy pregnant women were recruited as controls throughout the same time period.

### Detections

Blood samples were obtained after the diagnosis of ICP and before the administration of any medication or intervention. The serum SOD levels were determined using an ELISA commercial kit (#EIASODC, Thermo Fisher Scientific, Waltham, MA, USA) in accordance with the manufacturer's procedure. The assay's sensitivity was 0.044 U/mL, and samples and standards were examined in duplicate.

HBV serum marker HBsAg and other clinical parameters were detected at admission in the Department of Clinical Laboratory, The First Affiliated Hospital of Xi’an Jiaotong University.

### Statistical methods

Results are presented as means and standard deviations (SDs). Demographic characteristics were compared using the chi-square test or Fisher's exact test for categorical data, and the Wilcoxon rank sum test for continuous variables. The ability of serum SOD level to distinguish the ICP group from the control group was assessed using receiver operating characteristic (ROC) curves. The maximum of the sum of sensitivity and specificity was used to define cutoffs for continuous variables. Data were analyzed using SPSS version 16.0 software (IBM Corporation, Somers, NY, USA). Differences were considered to be of statistical significance when the *p* value was below 0.05.

## Results

### Baseline characteristics

The study cohort included 70 ICP patients and 70 healthy pregnant women. The clinical baseline characteristics of ICP patients and healthy controls (HC) are shown in Table 1.

**Table 1:** Laboratory parameters in patients with ICP

Characteristics	HC (*n* = 70)	ICP (*n* = 70)	*p* Value
Age (yr)	30.39 ± 0.47	30.76 ± 0.44	0.567
Gestational week at diagnosis (w)		32.83 ± 0.37	NA
Gestational week at delivery (w)	38.16 ± 0.12	36.72 ± 0.11	< 0.01
TBA (μmol/L)	6.15 ± 0.28	28.28 ± 1.44	< 0.01
SOD (U/L)	98.70 ± 2.95	243.24 ± 12.57	< 0.01
HBV-infection	18 (25.71%)	36 (51.53%)	< 0.05
AST (U/L)	23.59 ± 0.99	125.71 ± 11.37	< 0.01
ALT (U/L)	19.56 ± 1.08	118.56 ± 11.20	< 0.01
ALP (U/L)	38.76 ± 2.61	110.13 ± 8.21	< 0.01
GGT (U/L)	18.03 ± 1.31	18.74 ± 1.28	0.698
CHOL (mmol/L)	6.44 ± 0.23	5.03 ± 0.23	< 0.01
TBIL (μmol/L)	20.22 ± 0.82	25.56 ± 3.26	0.105
DBIL (μmol/L)	11.82 ± 0.58	16.18 ± 2.20	0.058
IDBIL (μmol/L)	8.40 ± 0.41	9.34 ± 1.10	0.433

*TBA* Total bile acid, *SOD* Superoxide dismutase, *AST* Aspartate transaminase, *ALT* Alanine transaminase, *ALP* Alkaline phosphatase, *GGT* Gamma glutamyl transferase, *CHOL* Cholesterol, *TBIL* Total bilirubin, *DBIL* Direct bilirubin, *IDBIL* Indirect bilirubin

The two groups were similar in maternal age, while the ICP group showed significantly higher serum levels of TBA, SOD, AST, ALT, ALP and CHOL. Fifty-one percent of the pregnant women in the ICP group and twenty-five the percent pregnant women in the control group were infected with HBV. The ICP group showed significantly earlier delivery compared with the control group.

### ICP is associated with high serum SOD levels

**Fig. 1:**
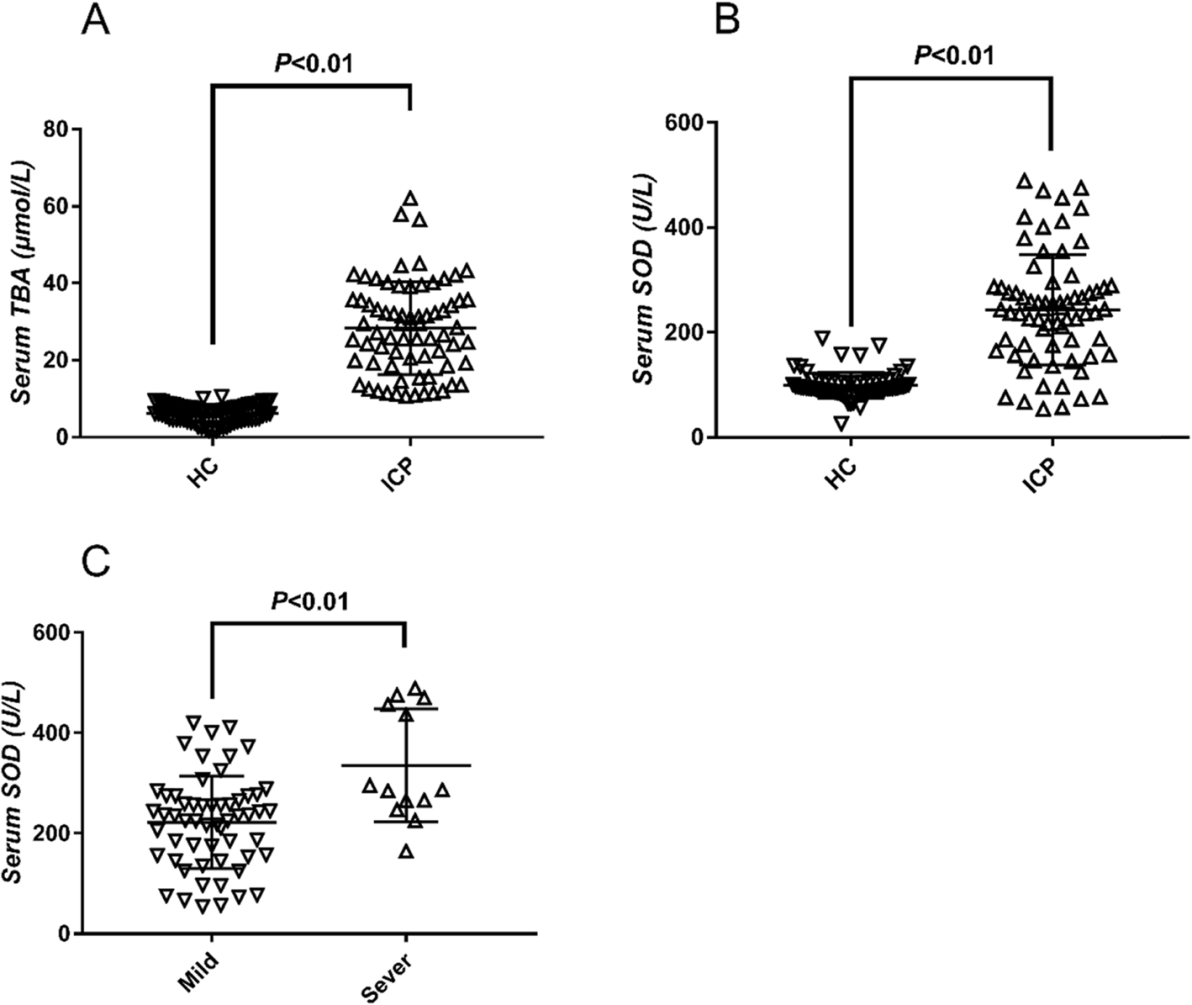
Serum SOD levels are increased in ICP patients. **A** TBA levels are higher in ICP patients compared with healthy pregnant women; **B** SOD levels are higher in ICP patients compared with healthy pregnant women; **C** SOD levels are higher in severe ICP patients compared with mild ICP patients

Compared with the control group, pregnant women in the ICP group had significantly higher levels of serum TBA (28.28 ± 1.44 μmol/L vs 6.15 ± 0.28 μmol/L, *p* < 0.01) and SOD (243.24 ± 12.57 U/L vs 98.70 ± 2.95 U/L, *p* < 0.01) (Fig. 1A and B).

We divided ICP patients into severe (TBA > 40 μmol/L, *n* = 13) and mild (10 < TBA < 40 μmol/L, *n* = 57) subgroups, and their characteristics are shown in Table 2. The severe and mild subgroups were similar in maternal age, while the severe subgroup showed significantly higher serum levels of SOD, AST, ALT, ALP, CHOL and TBIL. The severe subgroup showed significantly lower birth weight and delivery compared with the mild subgroup. Three cases of intrauterine fetal death (IUFD) occurred at 33, 32 and 34 weeks in the severe ICP subgroup.

**Table 2:** Laboratory parameters in patients with ICP (Severity)

Characteristics	ICP TBA < 40	ICP TBA ≥ 40	*p* Value
	(*n* = 57)	(*n* = 13)	
Intrauterine fetal death	0	3	NA
Age (yr)	30.61 ± 0.44	31.38 ± 1.39	0.501
Gestational week at diagnosis (w)	33.12 ± 0.36	31.65 ± 1.15	0.094
Gestational week at delivery (w)	36.87 ± 0.12	36.20 ± 0.25	< 0.05
Birth weight (g)	2964.65 ± 48.74	2749.10 ± 56.11	0.075
SOD (U/L)	222.15 ± 12.20	335.72 ± 31.19	< 0.01
HBV-infection	26 (45.61%)	10 (76.92%)	< 0.05
AST (U/L)	92.45 ± 8.12	271.51 ± 21.72	< 0.01
ALT (U/L)	92.94 ± 9.53	230.88 ± 27.14	< 0.01
ALP (U/L)	101.50 ± 7.95	148.00 ± 25.41	< 0.05
GGT (U/L)	18.02 ± 1.45	21.90 ± 2.57	0.240
CHOL (mmol/L)	4.578 ± 0.24	6.12 ± 0.47	< 0.05
TBIL (μmol/L)	19.00 ± 1.61	54.27 ± 13.07	< 0.01
DBIL (μmol/L)	11.83 ± 1.07	35.26 ± 9.46	< 0.01
IDBIL (μmol/L)	7.14 ± 0.76	19.01 ± 4.00	< 0.01

Compared with the mild ICP subgroup, the severe ICP subgroup had significantly higher levels of serum SOD (335.72 ± 31.19 U/L vs 222.15 ± 12.20 U/L, *p* < 0.01) (Fig. 1C) and a higher rate of HBV infection (76.92% vs 45.61%, *p* < 0.05).

### HBV infection promotes oxidative stress in ICP patients

We divided ICP patients into HBsAg-positive (*n* = 36) and HBsAg-negative (*n* = 34) subgroups, and their characteristics are shown in Table 3.

**Table 3:** Laboratory parameters in patients with ICP (HBV infection)

Characteristics	ICP HBsAg (-)	ICP HBsAg ( +)	*p* Value
	(*n* = 34)	(*n* = 36)	
Age (yr)	31.59 ± 0.59	29.97 ± 0.63	0.067
Gestational week at diagnosis (w)	33.85 ± 0.41	31.86 ± 0.56	< 0.01
Gestational week at delivery (w)	37.12 ± 0.14	36.30 ± 0.13	< 0.01
Birth weight (g)	3045.26 ± 64.26	2816.27 ± 51.03	< 0.01
TBA (μmol/L)	24.53 ± 1.78	31.81 ± 2.09	< 0.01
SOD (U/L)	196.65 ± 11.75	287.24 ± 19.21	< 0.01
AST (U/L)	95.32 ± 12.40	154.40 ± 17.61	< 0.01
ALT (U/L)	95.00 ± 14.50	140.81 ± 16.27	< 0.05
ALP (U/L)	105.23 ± 12.93	114.76 ± 10.40	0.565
GGT (U/L)	17.03 ± 1.67	20.36 ± 1.90	0.195
CHOL (mmol/L)	4.52 ± 0.28	5.51 ± 0.32	< 0.05
TBIL (μmol/L)	17.14 ± 2.39	33.49 ± 5.42	< 0.01
DBIL (μmol/L)	10.53 ± 1.28	21.53 ± 3.93	< 0.05
IDBIL (μmol/L)	6.62 ± 1.92	11.91 ± 1.73	< 0.05

The HBsAg-positive and HBsAg-negative subgroups were similar in maternal age, while the HBsAg-positive subgroup showed significantly higher serum levels of TBA, SOD, AST, ALT, CHOL, and TBIL. The HBsAg-positive subgroup showed significantly lower birth weight, and earlier diagnosis and delivery compared to HBsAg-negative subgroup.

**Fig. 2:**
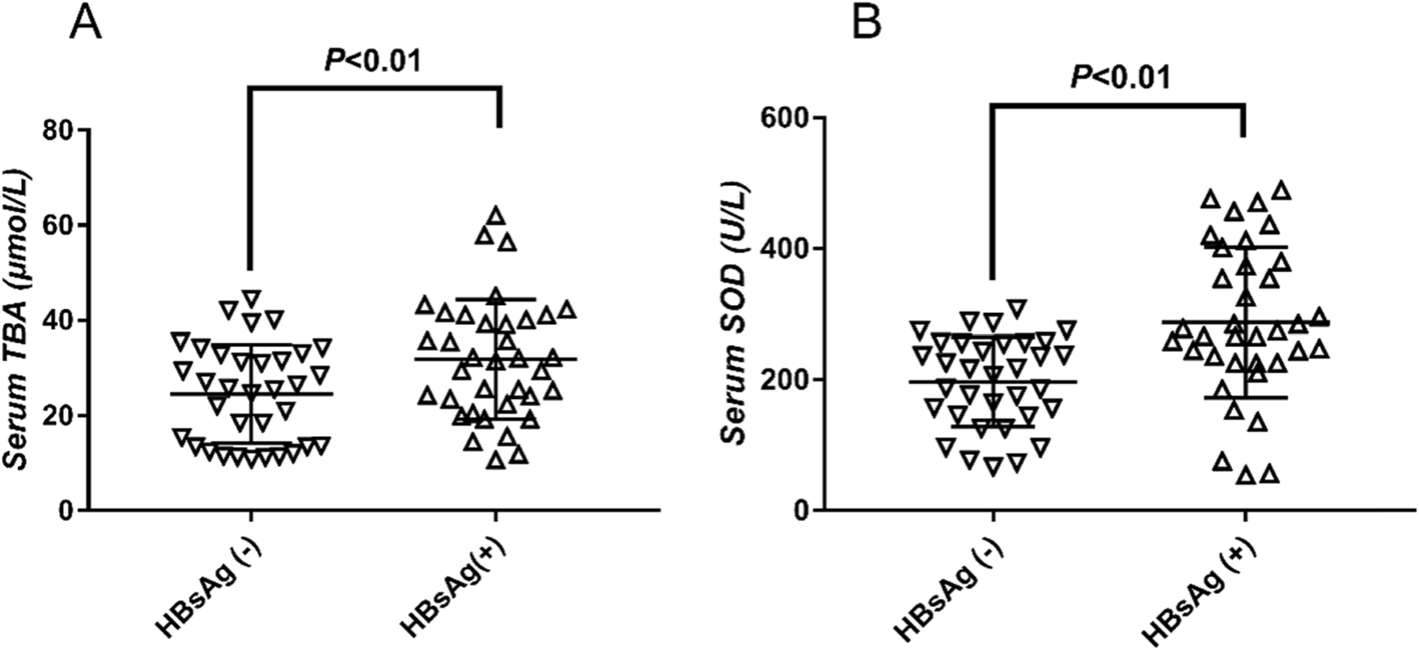
Serum SOD levels are increased in HBsAg-positive ICP patients. **A** TBA levels are higher in HBsAg-positive ICP patients compared with HBsAg-negative ICP patients; **B** SOD levels are higher in HBsAg-positive ICP patients compared with HBsAg-negative ICP patients

Compared with HBsAg-negative ICP patients, HBsAg-positive ICP patients had significantly higher levels of serum TBA (31.81 ± 2.09 μmol/L vs 24.53 ± 1.78 μmol/L, *p* < 0.01) and SOD (287.24 ± 19.21 U/L vs 196.65 ± 11.75 U/L, *p* < 0.01) (Fig. 2).

### The potential diagnostic value of serum SOD for ICP

We identified the potential risk factors for ICP using univariate and multivariate Cox regression analyses. As shown in Table 4, SOD (HR = 1.035, 95% CI: 1.023–1.048, *P* < 0.01), AST (HR = 1.165, 95% CI: 1.093–1.241, *P* < 0.01), ALT (HR = 1.093, 95% CI: 1.055–1.132, *P* < 0.01), ALP (HR = 1.041, 95% CI: 1.027–1.056, *P* < 0.01), and CHOL (HR = 0.671, 95% CI: 0.551–0.816, *P* < 0.01) were significantly associated with the diagnosis of ICP. We then performed a forward multivariate analysis. The results revealed that SOD (HR = 1.048, 95% CI: 1.017–1.081, *P* < 0.01) was an independent risk factor for the diagnosis of ICP.

**Table 4:** Uni-and multivariate logistic analysis of risk factors associated with the diagnosis of ICP

	Univariate			Multivariate		
	HR	95% CI	*P*	HR	95% CI	*P*
Age (yr)	1.026	0.940–1.120	0.564			
SOD (U/L)	1.035	1.023–1.048	< 0.01	1.048	1.017–1.081	< 0.01
AST (U/L)	1.165	1.093–1.241	< 0.01	1.115	0.933–1332	0.233
ALT (U/L)	1.093	1.055–1.132	< 0.01	1.049	0.944–1.165	0.371
ALP (U/L)	1.041	1.027–1.056	< 0.01	1.042	0.986–1.101	0.147
GGT (U/L)	1.006	0.976–1.038	0.696			
CHOL (mmol/L)	0.671	0.551–0.816	< 0.01	0.485	0.220–1.070	0.073
TBIL (μmol/L)	1.020	0.994–1.048	0.129			
DBIL (μmol/L)	1.044	0.999–1.090	0.058			
IDBIL (μmol/L)	1.020	0.971–1.073	0.426			

**Fig. 3:**
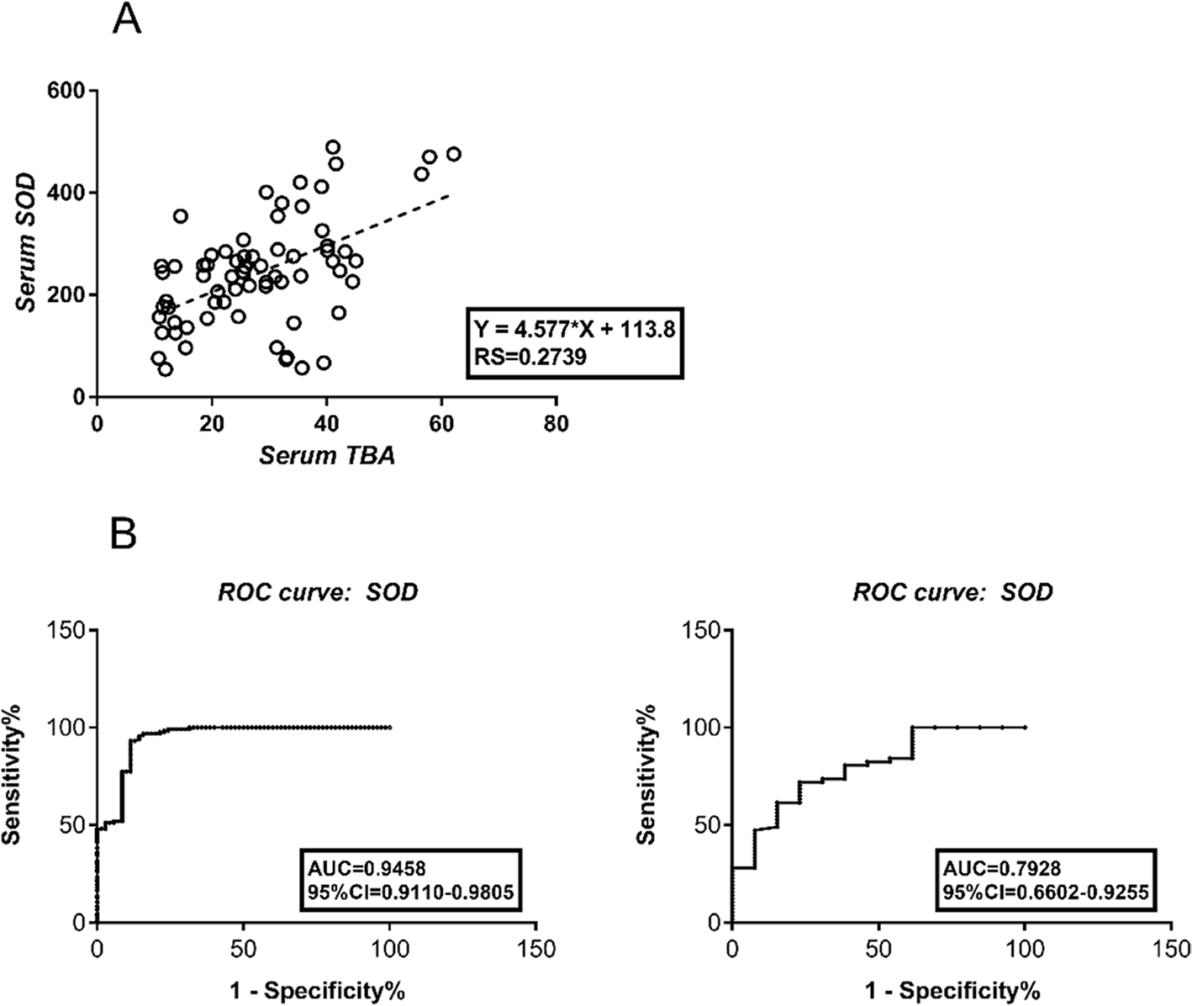
Correlations of serum SOD and TBA and diagnostic value of serum SOD in ICP patients. **A** Serum SOD is positively associated with TBA (RS = 0.2739, *p* < 0.01); **B** ROC curve for serum SOD (AUC: 0.9458, 95% CI: 0.9110–0.9805) (diagnosis); **C** ROC curve for serum SOD (AUC: 0.7928, 95% CI: 0.6602- 0.9255) (severity)

To evaluate the diagnostic value of serum SOD levels, the association between SOD and TBA levels was analyzed using Pearson correlation analysis. We showed that the level of serum SOD was positively associated with TBA (RS = 0.2739, *p* < 0.01) (Fig. 3A). The ROC curves of SOD showed strong separation between the ICP and control groups, with an area under the curve (AUC) of 0.9458 (95% CI 0.9110–0.9805). The maximum sensitivity and specificity for SOD as a predictor for ICP diagnosis were achieved at 121.4 U/mL (Fig. 3B), and the maximum sensitivity and specificity for SOD as a predictor for ICP severity were at 274.6 U/mL, with an AUC of 0.7928 (95% CI 0.6602- 0.9255) (Fig. 3C).

## Discussion

ICP, along with HBV infection, is one of the most frequent illnesses during pregnancy. It usually develops during the third trimester and is caused by the mother's aberrant bile acid metabolism. ICP can cause premature delivery, meconium-stained amniotic fluid, intrauterine fetal death, and postpartum hemorrhage of pregnant women [2]. In the present study, the ICP patients showed significantly earlier delivery compared with the healthy pregnant women, and three cases of IUFD occurred among the ICP patients.

In China, the incidence of HBV infection in pregnant women ranges from 2 to 8%. HBV infection increases the likelihood of developing ICP, and ICP paired with HBV infection frequently causes placental function defects, aggravation of cholestasis, significant hypoxia, fetal distress, fetal growth restriction, preterm birth or stillbirth, and other severe negative effects [11]. In the present study, we found that fifty-one percent pregnant women in the ICP group were infected by HBV, compared with twenty-five percent in the control group, and seventy-six percent ICP patients in the severe subgroup were HBV infected, compared with forty-five percent in the mild subgroup. These findings suggest that HBV testing should be performed frequently in patients with ICP, particularly in pregnant women with itchy skin who have been diagnosed with severe ICP. Moreover, we found that HBsAg-positive ICP patients had higher levels of serum TBA and SOD compared with HBsAg-negative patients. To avoid adverse pregnancy outcomes, we suggest that clinicians should strengthen the screening for ICP in pregnant women with HBV and improve the monitoring of serum bile acid and liver function in patients during pregnancy.

After finding a higher HBV infection rate in the ICP group compared with the control group, we wondered whether and how HBV infection participates in disease pathogenesis of ICP. Although the exact mechanism underlying the relationship between HBV infection and ICP progression is poorly understood, there are a few possible explanations. Several studies have linked HBV infection to oxidative stress, which has been shown to participate in the pathogenesis of ICP. The term "oxidative stress" refers to a discrepancy between the synthesis and clearance of reactive oxygen species, which leads to oxidative damage [12]. HBV is a hepatophilic virus that has been linked to the development of oxidative stress. It is believed that HBV generates oxidative stress by altering mitochondrial function and modulating host gene expression. The pathogenesis of many human diseases, including ICP, is influenced by oxidative stress. Severe oxidative stress is present in both human and mouse livers with ICP, and there is increasing evidence that oxidative stress induced by bile acids is closely associated with the pathogenesis of ICP [13]. Accumulation of bile acids could disrupt cell membranes through their detergent action and cause mitochondrial respiratory dysfunction, leading to the generation and accumulation of free oxygen radicals in mitochondria, which in turn oxidatively modify lipids, proteins, and nucleic acids, and cause hepatocyte apoptosis [14, 15]. All these changes lead to the impaired hepatic function and elevation in serum ALT and AST levels. A recent study evaluated the thiol/disulfide ratio, an indicator of oxidative stress, to explore whether it may reveal oxidative stress in a group of women with ICP; it found a considerably lower native and total thiol ratio, as well as significantly greater disulfide levels [16]. MgSO_4_ has a long history of being used to improve maternal and fetal outcomes. During pregnancy, the placenta plays a fundamental role in maternal–fetal mutual exchange of oxygen and nutrients. A recent study has found that MgSO_4_ offeres protection against oxidative damage in the ICP model rat placenta [17]. Due to the central role of oxidative stress in ICP disease progression, increased oxidative stress during ICP disease progression on the background of HBV infection resulted in significantly higher oxidative stress, as well as lower birth weight, earlier diagnosis, and earlier delivery compared with HBsAg-negative ICP patients.

The pathogenesis of various human diseases has been linked to an imbalance between systemic oxidative stress and detoxification mechanisms. However, there has not been much research on the diagnostic and predictive roles of oxidative markers in ICP. Hu et al. has recently reported considerably decreased blood levels of the 8-epimer of prostaglandin F2 alpha and glutathione peroxidase in patients with ICP, which has been attributed to human antioxidant systems failing to regulate homeostasis in ICP patients [8]. Ozler et al. investigated the total antioxidant status (TAS) and the total oxidative stress (TOS) levels in ICP. They found increased TAS, TOS, and oxidative stress index, which may be the consequences of augmented inflammatory mechanisms [18]. SOD is a key endogenous antioxidant enzyme and protects cells from intracellular and extracellular oxidative damage. It reduces the harmful effects of ROS by converting damaging superoxide to hydrogen peroxide [19]. In the present study, we evaluated the levels of serum SOD, which increases as an adaptive response to the elevated systemic oxidative stress during ICP. We found that the circulating SOD level was markedly elevated in the ICP patients compared with the healthy pregnant women. The serum SOD level was also associated with the disease severity; SOD > 121.4 U/mL might serve to predict the ICP diagnosis, and SOD > 274.6 U/mL might serve to predict the ICP severity. This present study suggested that the serum SOD level could serve as an independent predictor of mortality in ICP patients, and very early testing of the serum SOD level may be a diagnostic and predictive marker.

There are some limitations to the present study. First, the study was designed as a cross-sectional study, which included a limited number of patients. Second, the current study has limitations intrinsic to retrospective research, such as possible biases, including selection bias.

## Conclusion

We found a higher level of serum SOD in patients with ICP, which was mostly due to the increased oxidative stress throughout the disease development. HBV infection increased oxidative stress and contributed to the development of ICP. Serum SOD levels might be utilized to predict ICP severity and diagnosis. Furthermore, developing techniques to reduce oxidative stress might reveal new insights into pathogenetic mechanisms, making it a possible focus for treating ICP patients.

## Data Availability

The datasets used and/or analyzed during the current study are available from the corresponding author on reasonable request.

## Abbreviations

ICP: Intrahepatic cholestasis of pregnancy
TBA: Total bile acid
HBV: Hepatitis B virus
SOD: Superoxide dismutase
CHB: Chronic hepatitis B
IUFD: Intrauterine fetal death
AUC: Area under the curve
TAS: Total antioxidant status
TOS: Total oxidative stress
